# Skin sodium content as a predictor of blood pressure response to renal denervation

**DOI:** 10.1038/s41440-023-01450-4

**Published:** 2023-10-25

**Authors:** Merve Guenes-Altan, Axel Schmid, Dennis Kannenkeril, Peter Linz, Christian Ott, Agnes Bosch, Mario Schiffer, Michael Uder, Roland E. Schmieder

**Affiliations:** 1 Department of Nephrology and Hypertension, University Hospital Erlangen, Friedrich-Alexander University Erlangen-Nuremberg (FAU), Erlangen, Germany; 2 Institute of Radiology, University Hospital Erlangen, Friedrich-Alexander University Erlangen-Nuremberg (FAU), Erlangen, Germany

**Keywords:** Renal denervation, Tissue sodium, Sodium MRI

## Abstract

Patients with treatment resistant hypertension (TRH) are known to have elevated sodium (Na) content in muscle and skin. Renal denervation (RDN) emerged as an adjacent therapeutic option in this group of patients. This analysis aimed at evaluating whether tissue Na content predicts blood pressure (BP) response after RDN in patients with TRH. Radiofrequency-device based RDN was performed in 58 patients with uncontrolled TRH. Office and 24-h ambulatory BP were measured at baseline and after 6 months. To assess tissue Na content Na magnetic resonance imaging (Na-MRI) was performed at baseline prior to RDN. We splitted the study cohort into responders and non-responders based on the median of systolic 24-h ambulatory blood pressure (ABP) reduction after 6 months and evaluated the association between BP response to RDN and tissue Na content in skin and muscle. The study was registered at http://www.clinicaltrials.gov (NCT01687725). Six months after RDN 24-h ABP decreased by −8.6/−4.7 mmHg. BP-Responders were characterized by the following parameters: low tissue sodium content in the skin (*p* = 0.040), female gender (*p* = 0.027), intake of aldosterone antagonists (*p* = 0.032), high baseline 24-h night-time heart rate (*p* = 0.045) and high LDL cholesterol (*p* < 0.001). These results remained significant after adjustment for baseline 24-h systolic BP. Similar results were obtained when the median of day-time and night-time ABP reduction after 6 months were used as cut-off criteria for defining BP response to RDN. We conclude that in addition to clinical factors including baseline 24-h ABP Na-MRI may assist to select patients with uncontrolled TRH for RDN treatment.

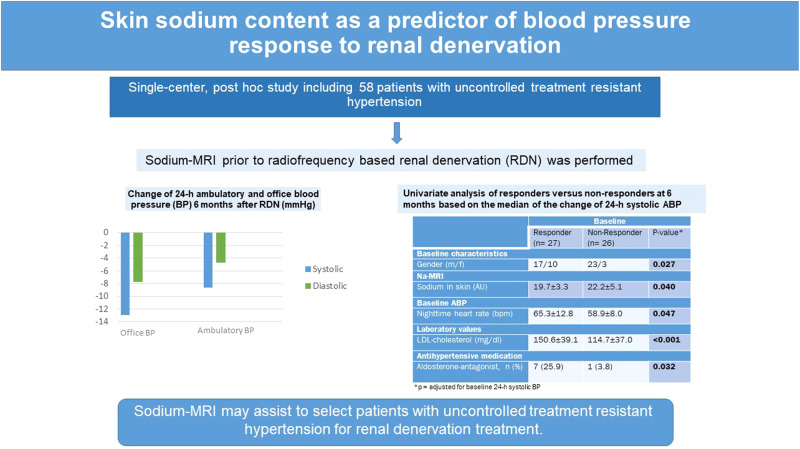

## Introduction

Arterial hypertension (HTN) is highly prevalent worldwide and a major risk factor for cardiovascular disease (CVD) and stroke. In 2016, it was estimated that 46.0% of adults in the United States suffered from HTN [1]. A multinational study in adults revealed that 55.6% of patients were aware of their diagnosis with only 17.1% having adequately controlled HTN [2]. Furthermore, the prevalence of treatment resistant hypertension (TRH) among patients with HTN was approximately 10% [3].

Increased activity of the sympathetic nervous system (SNS) activity and increased sodium (Na) retention have been found to play an important role in the pathogenesis of TRH while both mechanisms are interconnected to each other [4–6]. High SNS activity results in increased Na and water retention [7, 8]. This enhances vasoconstriction due to vasoactive hormones and induces hypertrophic response of the myocardium and vessels [9]. Thus, targeting increased SNS activity appears to be an attractive treatment strategy [10].

The ESH 2021 and ESC 2023 consensus statements recommend RDN as an adjacent treatment option to achieve BP control in patients with uncontrolled TRH in addition to pharmacotherapy and lifestyle changes [11, 12]. Since blood pressure (BP) response after RDN has a large variability and there exist patients with poor BP response after RDN, the need to identify determinants of BP response to RDN have been underlined in the consensus statements [11, 12].

Several studies have documented an accumulation of Na without simultaneously commensurate water which leads to the concept that there must exist non-osmotic storage of Na in tissue, for example in skin and muscle [13–16]. Na magnetic resonance imaging (Na-MRI) can be used to visualize and quantify Na content in tissue. Using this non-invasive technique elevated Na content in muscle and skin has been detected in patients with hypertension and in particular in patients with TRH [17]. Moreover, we previously analyzed the impact of RDN on tissue Na content in patients with treatment resistant hypertension and did not observe any change in muscle or skin Na content 6 months after RDN [18]. The objective of the present study was to analyze whether tissue Na content in patients with TRH serves as a determinant of BP response in RDN.

## Methods

### Study design

Our single-center, post-hoc study includes 58 patients with uncontrolled TRH who underwent RDN and Na-MRI. The patients participated in the “Renal Denervation in Treatment Resistant Hypertension” trial, an investigator initiated study program performed only in our Erlanger center. The study was registered at http://www.clinicaltrials.gov (NCT01687725). All patients were followed up for 6 months at the Clinical Research Centre of the Department of Nephrology and Hypertension, University Hospital Erlangen-Nuremberg, Germany (www.crc-erlangen.de).

The respective study protocol was approved by the local Ethical Review Committee (ethics committee of the University of Erlangen-Nuremberg) and the study was conducted according to the Declaration of Helsinki and Good Clinical Practice. Written informed consent was obtained from all patients and prior to study inclusion.

### Study cohort

All patients who participated in our study were aged 40–77 years, had uncontrolled TRH with 3–10 antihypertensive drugs and were eligible for Na-MRI examination without MRI contraindication. True hypertension was confirmed by 24-h ambulatory blood pressure (ABP) measurement (average 24-h ABP ≥ 130/80 mmHg). All patients fulfilled the following exclusion criteria: No known secondary cause of HTN including hyperaldosteronism, no significant renal artery pathologies, no prior RDN and no known contraindication for RDN procedure (e.g. renal artery stenosis > 50%, implanting of renal stents).

### Assessments

Baseline assessments included office and 24-h ABP measurements, Na-MRI examination, collection of demographic data and antihypertensive medication as well as physical examination, standard blood and urine tests. Office and ambulatory BP were measured with validated devices following the recommendations of the European Society of Hypertension/European Society of Cardiology [19, 20]. Office BP was assessed after a rest of at least 5 min and repeated twice in a sitting position with a validated automatic device. Ambulatory BP was conducted with a validated device (Mobilograph, IEM, Aachen, Germany) and mean values for 24-h, day-time and night-time ABP were calculated according to the published recommendations. Estimated glomerular function (eGFR) was calculated using the Chronic Kidney Disease Epidemiology Collaboration (CKD-EPI) formula [21]. Adverse events occurring during the trial were recorded at each visit.

### RDN procedure

A radiofrequency-based Symplicity-Flex catheter (Symplicity by Ardian Inc, Palo Alto, CA, USA) was used for RDN procedure. A renal angiogram was previously performed to exclude renal artery abnormalities. As previously described [22], the femoral artery was accessed with standard endovascular technique and renal arteries of both sides were treated in one session. Up to 6 radiofrequency ablations (energy delivery for up to 120 s and 8 watts each) were applied longitudinally and rotationally within each artery to achieve a full 4-quadrant ablation. Visceral pain during the procedure was managed with anxiolytics and narcotics and patients were given 500 IE heparin.

### Na-MRI measurements

Skin and muscle Na content in the left lower leg were measured non-invasively with a clinical 3.0 T MR system (Magnetom Skyra, Siemens Healthineers, Erlangen, Germany) using a transit/receive Na RF birdcage knee coil (32.6 MHz, Stark Contrast, Erlangen, Germany) at baseline and 6 months after RDN. A detailed description of the procedure, as well as reliability and accuracy have been shown previously [9, 23].

### Statistical analysis

Statistical analysis was performed using SPSS Statistics 28.0 (IBM, Armonk, NY, USA) and data were expressed as mean ± standard deviation (SD) in text and tables. Paired *t*-test was applied for the comparison of 6 months follow-up BP data versus baseline. Predictors of BP change were assessed by comparing responders versus non-responders defined by the median reduction of 24-h, day-time and night-time systolic ABP at 6 month follow-up visit, respectively. Bivariate correlation analyses were assessed by performing Pearson’s test. Subsequently, since baseline BP predicted the BP change in many previous RDN studies related to Wilders principle “law of initial value” [24], we adjusted our univariate approach only to 24-h, day-time and night-time systolic ABP. A two-sided *p* value of <0.05 was considered statistically significant.

## Results

### Clinical characteristics

We included 58 patients in our study with a mean age of 62 years. Most patients were male and overweight. About half of the patients had type 2 diabetes (T2D). All patients had uncontrolled TRH with 6.2 (3.0–10.0) antihypertensive drugs on average. Four patients were not on diuretic therapy because of contraindications and drug intolerance. The detailed clinical characteristics are shown in Table 1.

**Table 1:** Clinical characteristics

Demographic data	
Age (years)	61.6 ± 9.7
Male/female (*n*/*n*)	37/14
Body mass index (kg/m^2^)	30.2 ± 3.9
Weight (kg)	90.0 ± 15.6
Comorbidities	
Diabetes mellitus, *n* (%)	32 (55)
Coronary artery disease, *n* (%)	20 (35)
Left ventricular hypertrophy, *n* (%)	15 (26)
Hyperlipidaemia, *n* (%)	24 (41)
History of stroke, TIA, *n* (%)	9 (16)
Current smoking, *n* (%)	9 (16)
Na-MRI	
Sodium content in M. triceps surae (AU)	20.6 ± 4.4
Sodium content in skin (AU)	20.9 ± 4.3
Office BP	
Office systolic BP (mmHg)	158 ± 23
Office diastolic BP (mmHg)	87 ± 16
Office heart rate (bpm)	71 ± 15
24-h ABP	
24-h systolic ABP (mmHg)	157 ± 16
24-h diastolic ABP (mmHg)	87 ± 13
24-h ambulatory heart rate (bpm)	67 ± 12
Day-time systolic ABP (mmHg)	159 ± 16
Day-time diastolic ABP (mmHg)	89 ± 13
Day-time heart rate (bpm)	69 ± 12
Night-time systolic ABP (mmHg)	151 ± 21
Night-time diastolic ABP (mmHg)	80 ± 13
Night-time heart rate (bpm)	62 ± 11
Systolic nocturnal dipping rate (%)	5.1 ± 11.0
Diastolic nocturnal dipping rate (%)	9.9 ± 10.1
Laboratory values	
HbA1c (%)	6.6 ± 1.3
Creatinine (mg/dl)	1.2 ± 0.5
eGFR, CKD-Epi formula (mL/min/1.73 m²)	69.5 ± 24.7
Triglyceride	207.2 ± 117.5
Cholesterol (mg/dl)	196.0 ± 52.0
LDL-cholesterol (mg/dl)	131.5 ± 42.0
Hemoglobin (g/dl)	13.9 ± 1.6
Hematocrit (%)	40.6 ± 4.4
Antihypertensive medication	
Number of antihypertensive medication, n (%)	6.2 ± 1.6
ACE inhibitors, *n* (%)	20 (35)
ARBs, *n* (%)	45 (78)
Direct renin inhibitors, *n* (%)	25 (43)
Betablockers, *n* (%)	46 (79)
Calcium-channel blockers, *n* (%)	49 (85)
Diuretics, *n* (%)	54 (93)
Aldosterone antagonists, *n* (%)	10 (17)
Vasodilators, *n* (%)	21 (36)
Centrally acting sympatholytics, *n* (%)	46 (79)

Data are presented as mean ± SD

*Na* Sodium, *Na-MRI* Na magnetic resonance imaging, *BP* blood pressure, bpm beats per minute, *ABP* ambulatory blood pressure, *BMI* body mass index, *LDL* low density lipid, HbA1c glycated hemoglobin, *eGFR* estimated glomerular filtration rate, *CKD-EPI* Chronic Kidney Disease Epidemiology Collaboration, *ACE* angiotensin-converting enzyme, *ARB* angiotensin receptor blocker

### Blood pressure

The average 24-h ABP in our study cohort was 157/87 mmHg and the office BP was 158/87 mmHg. Six months after RDN 24-h ABP was reduced by −8.6/−4.7 mmHg and office BP was reduced by −12.9/−7.7 mmHg (all *p* < 0.001, see Table 2).

**Table 2:** Change of 24-h ambulatory and office blood pressure 6 months after renal denervation (*n* = 53)

BP change	6 months
Office systolic BP (mmHg)	−12.9 ± 21.1*
Office diastolic BP (mmHg)	−7.7 ± 12.5*
Office heart rate (bpm)	−3.2 ± 10.0*
24-h systolic ABP (mmHg)	−8.6 ± 14.0*
Day-time systolic ABP (mmHg)	−8.6 ± 14.3*
Night-time systolic ABP (mmHg)	−10.3 ± 22.6*
24-h diastolic ABP (mmHg)	−4.7 ± 9.5*
Day-time diastolic ABP (mmHg)	−4.7 ± 12.5*
Night-time diastolic ABP (mmHg)	−4.6 ± 12.5*
24-h ambulatory heart rate (bpm)	1.1 ± 6.1
Day-time ambulatory heart rate (bpm)	1.4 ± 6.7
Night-time ambulatory heart rate (bpm)	0.5 ± 6.5

Data are presented as mean ± SD

*BP* blood pressure, *ABP* ambulatory blood pressure, *bpm* beats per minute

*p < 0.05 versus baseline

Medication change (conducted according to the discretion of the primary care physician) did not differ between the two groups (*p* = 0.320): At 6 months, decrease in number of antihypertensive drugs took place in 37.0% of responders versus 23.1% in non-responders, whereas increase in medication number occurred in 25.9% in responders and 23.1% in non-responders.

### Na-MRI measurements

At baseline, skin Na content was 20.9 ± 4.3 AU and muscle Na content was 20.6 ± 4.4 AU. Six months after RDN we observed no change in skin (20.9 ± 4.3 AU versus baseline 21.1 ± 4.9 AU; *p* = 0.915) and muscle (20.6 ± 4.4 AU versus baseline 20.9 ± 4.0 AU; *p* = 0.683) Na content.

### Correlation analysis

**Fig. 1:**
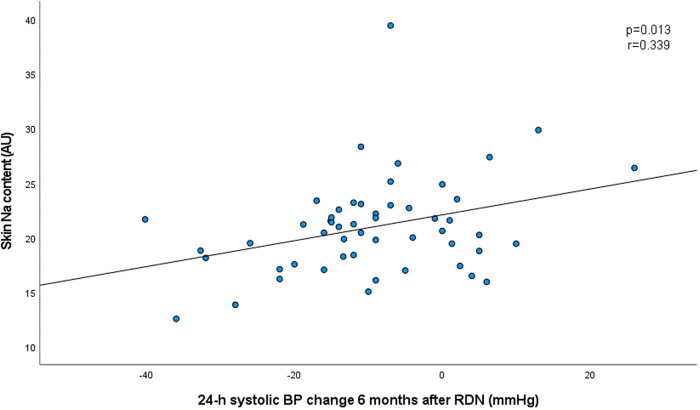
Correlation between skin sodium content at baseline and 24-h systolic ambulatory blood pressure change 6 months after renal denervation

We observed a correlation between baseline 24-h systolic ABP and BP response 6 months after RDN (*r* = −0.394, *p* = 0.003). In addition, we observed a correlation between skin sodium content and BP response (*r* = 0.339, *p* = 0.013, Fig. 1). Other than that, we did not observe any correlation between BP response and other parameters (all *p* > 0.1). In particular, no correlation was observed with gender, LDL-cholesterol, aldosterone-antagonist medication or night-time heart rate. We also did not observe any correlation between baseline 24-h systolic ABP and skin Na content (*r* = −0.070, *p* = 0.601).

### Predictors of BP response

To identify any predictors of BP response after RDN we splitted the study cohort according to the median systolic 24-h ABP reduction after 6 months into responders and non-responders (median ≤ −10.0 mmHg versus > −10.0 mmHg, Table 3). We identified patients with low skin Na, high BMI, female patients, patients with high LDL-cholesterol, medication with aldosterone antagonists and diuretics, and high night-time heart rate to be more likely to respond to RDN with >10 mmHg fall in 24-h ABP after 6 months. After adjustment for baseline 24-h systolic ABP, skin Na, gender, LDL-cholesterol, night-time heart rate and aldosterone-antagonist medication remained significantly different between responders and non-responders. (Table 3)

**Table 3:** Univariate analysis of responders versus non-responders at 6 months based on the median of the change of 24-h systolic ABP

	Baseline			
	Median ≤ −10 mmHg	Median > −10 mmHg	*P* value	*P* value*
	Responder (*n* = 27)	Non-Responder (*n* = 26)		
Change of systolic ABP at 6 months (mmHg)	−18.7 ± 8.4	0.2 ± 8.2	<0.001	**<0.001**
Baseline characteristics				
BMI (kg/m^2^)	31.2 ± 4.2	28.8 ± 3.3	0.024	0.066
Gender (m/f)	17/10	23/3	0.031	**0.027**
Age (years)	60.4 ± 10.4	62.7 ± 8.4	0.386	0.389
Na-MRI				
Sodium in M. triceps surae (AU)	19.5 ± 3.3	21.6 ± 5.3	0.095	0.111
Sodium in skin (AU)	19.7 ± 3.3	22.2 ± 5.1	0.042	**0.040**
Baseline office BP				
Systolic BP (mmHg)	156.5 ± 16.9	152.2 ± 23.2	0.448	
Diastolic BP (mmHg)	88.1 ± 16.6	83.6 ± 12.2	0.268	
Heart rate (bpm)	73.1 ± 13.9	65.6 ± 14.0	0.059	0.086
Baseline ABP				
24-h systolic BP (mmHg)	158.9 ± 12.5	151.8 ± 15.8	0.077	
24-h diastolic BP (mmHg)	89.2 ± 12.9	83.7 ± 10.1	0.090	
24-h heart rate (bpm)	69.2 ± 12.3	64.2 ± 9.8	0.121	0.124
Systolic day-time BP (mmHg)	161.1 ± 13.0	154.3 ± 15.5	0.091	
Diastolic day-time BP (mmHg)	91.8 ± 12.9	86.2 ± 10.3	0.089	
Heart rate day-time (bpm)	70.4 ± 11.7	65.8 ± 10.6	0.162	0.159
Systolic night-time BP (mmHg)	153.7 ± 17.1	145.2 ± 24.3	0.145	
Diastolic night-time BP (mmHg)	83.1 ± 13.4	77.0 ± 12.7	0.099	
Night-time heart rate (bpm)	65.3 ± 12.8	58.9 ± 8.0	0.045	**0.047**
Laboratory values				
Hematocrit (%)	41.9 ± 3.8	39.6 ± 4.9	0.059	0.102
LDL-cholesterol (mg/dl)	150.6 ± 39.1	114.7 ± 37.0	0.001	**<0.001**
Antihypertensive medication				
Diuretics, *n* (%)	23 (85.2)	26 (100)	0.041	0.053
Aldosterone-antagonist, *n* (%)	7 (25.9)	1 (3.8)	0.025	**0.032**
ACE inhibitors, *n* (%)	10 (37)	9 (35)	0.854	0.699
ARBs, *n* (%)	21(78)	21 (81)	0.788	0.691
Direct renin inhibitors, *n* (%)	14 (52)	8 (31)	0.119	0.182
Betablockers, *n* (%)	21 (78)	21 (81)	0.788	0.971
Calcium-channel blockers, *n* (%)	23 (85)	22 (85)	0.954	0.896
Vasodilators, *n* (%)	9 (33)	10 (38)	0.697	0.608
Centrally acting sympatholytics, *n* (%)	21 (78)	20 (77)	0.941	0.824

Data are presented as mean ± SD. Bold values represent *p*-values ≤ 0.05

*Na* sodium, *Na-MRI* Na magnetic resonance imaging, *BP* blood pressure, *bpm* beats per minute, *ABP* ambulatory blood pressure, *BMI* body mass index, *LDL* low density lipid, *ACE* angiotensin-converting enzyme, *ARB* angiotensin receptor blocker

*p = adjusted for baseline 24-h systolic BP

We repeated the whole analysis by dividing patients into 2 groups based on the median of day-time and night-time BP change after 6 months, respectively. Besides systolic ABP, skin Na, aldosterone-antagonist medication, baseline 24-h, day-time and night-time heart rate remained significantly different between responders and non-responders (Tables 4 and 5). Age, body mass index (BMI) and renal function (i.e. eGFR) were not identified as determinants of BP response. Thus, lower skin sodium content (together with a non-significant signal for muscle sodium content) emerged as a BP independent predictor of BP fall after RDN.

**Table 4:** Univariate analysis of responders versus non-responders at 6 months based on the median of the change of day-time systolic ABP

	Baseline			
	Median ≤ −12 mmHg	Median > −12 mmHg	*P* value	*P* value*
	Responder (*n* = 27)	Non-Responder (*n* = 26)		
Change of day-time systolic BP at 6 months (mmHg)	−19.7 ± 7.6	1.8 ± 9.1	<0.001	**<0.001**
Baseline characteristics				
BMI (kg/m^2^)	30.7 ± 4.2	29.3 ± 3.7	0.182	0.392
Gender (m/w)			0.389	0.256
Age (years)	59.5 ± 10.1	63.7 ± 8.2	0.106	0.089
Na-MRI				
Sodium in M. triceps surae (AU)	19.5 ± 3.0	21.6 ± 5.5	0.074	0.081
Sodium in skin (AU)	19.6 ± 2.6	22.4 ± 5.4	0.021	**0.015**
Baseline office BP				
Systolic BP (mmHg)	152.4 ± 17.4	156 ± 22.8	0.481	
Diastolic BP (mmHg)	87.1 ± 16.3	84.7 ± 12.9	0.550	
Heart rate (bpm)	73.2 ± 14.5	65.6 ± 13.3	0.056	0.150
Baseline ABP				
24-h systolic BP (mmHg)	159.8 ± 13.5	150.8 ± 14.5	0.023	
24-h diastolic BP (mmHg)	89.2 ± 12.1	83.6 ± 11.0	0.081	
24-h heart rate (bpm)	70.5 ± 12.1	63.0 ± 9.2	0.019	**0.025**
Systolic day-time BP (mmHg)	162.5 ± 13.8	152.8 ± 13.9	0.014	
Diastolic day-time BP (mmHg)	91.9 ± 12.1	86.1 ± 11.2	0.074	
Heart rate day-time (bpm)	71.8 ± 12.0	64.5 ± 9.6	0.025	**0.030**
Systolic night-time BP (mmHg)	153.6 ± 19.8	145.3 ± 22.1	0.153	
Diastolic night-time BP (mmHg)	82.6 ± 13.4	77.5 ± 13.0	0.159	
Night-time heart rate (bpm)	66.1 ± 12.2	58.1 ± 8.2	0.010	**0.012**
Laboratory values				
Hematocrit (%)	40.9 ± 4.1	40.7 ± 4.9	0.883	0.759
Triglyceride	233.6 ± 119.2	76.7 ± 104.7	0.071	0.071
Cholesterol (mg/dl)	204.3 ± 43.9	189.4 ± 58.7	0.299	0.286
LDL-cholesterol (mg/dl)	140.6 ± 36.0	125.0 ± 46.4	0.178	0.161
Antihypertensive medication				
Diuretics, *n* (%)	24 (88.9)	25 (96.2)	0.317	0.432
Aldosterone-antagonist, *n* (%)	7 (25.9)	1 (3.8)	0.025	**0.036**
ACE inhibitors, *n* (%)	10 (37.0)	9 (34.6)	0.854	0.583
ARBs, *n* (%)	21 (77.8)	21 (80.8)	0.788	0.681
Direct renin inhibitors, *n* (%)	14 (51.9)	8 (30.8)	0.119	0.201
Betablockers, *n* (%)	20 (74.1)	22 (84.6)	0.344	0.596
Calcium-channel blockers, *n* (%)	23 (85.2)	22 (84.6)	0.954	0.961
Vasodilators, *n* (%)	11 (40.7)	8 (30.8)	0.449	0.619
Centrally acting sympatholytics, *n* (%)	21 (77.8)	20 (76.9)	0.941	0.746

Data are presented as mean ± SD. Bold values represent *p*-values ≤ 0.05

*Na* sodium, *Na-MRI* Na magnetic resonance imaging, *BP* blood pressure, bpm beats per minute, *ABP* ambulatory blood pressure, *BMI* body mass index, *LDL* low density lipid, *ACE* angiotensin-converting enzyme, *ARB* angiotensin receptor blocker

*p = adjusted for baseline 24-h day-time systolic BP

**Table 5:** Univariate analysis of responders versus non-responders at 6 months based on the median of the change of night-time systolic ABP

	Baseline			
	Median ≤ −13 mmHg	Median > −13 mmHg	*P* value	*P* value*
	Responder (*n* = 27)	Non-Responder (*n* = 26)		
Change of night-time systolic BP at 6 months (mmHg)	−26.6 ± 11.1	3.2 ± 11.9	<0.001	**<0.001**
Baseline characteristics				
BMI (kg/m^2^)	30.5 ± 4.6	29.5 ± 3.2	0.338	0.931
Gender (m/w)	18/9	22/4	0.129	0.150
Age (years)	60.7 ± 10.0	62.4 ± 8.9	0.524	0.351
Na-MRI				
Sodium in M. triceps surae (AU)	19.8 ± 3.5	21.2 ± 5.3	0.257	0.251
Sodium in skin (AU)	19.7 ± 3.2	22.2 ± 5.1	0.038	**0.016**
Baseline office BP				
Systolic BP (mmHg)	157.3 ± 21.1	151.4 ± 19.1	0.290	
Diastolic BP (mmHg)	89.4 ± 13.5	82.3 ± 15.1	0.078	
Heart rate(bpm)	71.3 ± 12.8	67.6 ± 15.9	0.350	0.133
Baseline ABP				
24-h systolic BP (mmHg)	160.7 ± 13.2	149.9 ± 14.0	0.005	
24-h diastolic BP (mmHg)	90.4 ± 11.9	82.4 ± 10.5	0.011	
24-h heart rate (bpm)	69.9 ± 11.2	63.6 ± 10.6	0.053	**0.004**
Systolic day-time BP (mmHg)	160.4 ± 13.3	155.0 ± 15.6	0.183	
Diastolic day-time BP (mmHg)	91.4 ± 11.9	86.7 ± 11.7	0.148	
Heart rate day-time (bpm)	70.6 ± 10.9	65.5 ± 11.3	0.117	**0.017**
Systolic night-time BP (mmHg)	159.9 ± 17.8	138.8 ± 19.1	<0.001	
Diastolic night-time BP (mmHg)	86.6 ± 12.5	73.4 ± 10.7	<0.001	
Night-time heart rate (bpm)	65.7 ± 11.6	58.5 ± 9.3	0.021	**0.003**
Laboratory values				
eGFR, CKD-EPI (ml/min/1.73 m²)	74.9 ± 25.3	62.8 ± 21.2	0.065	0.129
Hematocrit (%)	41.1 ± 3.5	40.4 ± 5.3	0.574	0.668
Triglyceride	218.8 ± 125.8	192.1 ± 103.0	0.403	0.291
Cholesterol (mg/dl)	201.9 ± 45.8	191.9 ± 57.8	0.487	0.265
LDL-cholesterol (mg/dl)	137.6 ± 34.7	128.2 ± 48.3	0.416	0.165
Antihypertensive medication				
Diuretics, *n* (%)	24 (88.9)	25 (96.2)	0.317	0.303
Aldosterone-antagonist, *n* (%)	5 (18.5)	3 (11.5)	0.478	0.443
ACE inhibitors, *n* (%)	10 (37.0)	9 (34.6)	0.854	0.720
ARBs, *n* (%)	21 (77.8)	21 (80.8)	0.788	0.431
Direct renin inhibitors, *n* (%)	13 (48.1)	9 (34.6)	0.318	0.409
Betablockers, *n* (%)	19 (70.4)	23 (88.5)	0.104	0.124
Calcium-channel blockers, *n* (%)	22 (81.5)	23 (88.5)	0.478	0.747
Vasodilators, *n* (%)	8 (29.6)	11 (42.3)	0.336	0.243
Centrally acting sympatholytics, *n* (%)	22 (81.5)	19 (73.1)	0.465	0.841

Data are presented as mean ± SD. Bold values represent *p*-values ≤ 0.05

*Na* sodium, *Na-MRI* Na magnetic resonance imaging, *BP* blood pressure, bpm beats per minute, *ABP* ambulatory blood pressure, *BMI* body mass index, *LDL* low density lipid, *ACE* angiotensin-converting enzyme, *ARB* angiotensin receptor blocker

*p = adjusted for baseline 24-h night-time systolic BP

## Discussion

In our study we performed a post-hoc analysis of 58 patients with uncontrolled TRH who underwent RDN and Na-MRI. We observed a reduction of office BP by −12.9/−7.7 mmHg and 24-h ABP by −8.6/−4.7 mmHg 6 months after RDN. Considering BP reduction, our results are consistent with the results of the Global Symplicity Registry. In this worldwide registry, office BP decreased by −11.6/−4.3 mmHg and 24-h ABP decreased by −6.6/−3.9 mmHg 6 months after RDN [25].

From a clinical perspective, it is important to identify potential predictors for the efficacy of RDN procedure as guidance to personalize treatment options in hypertension. Many studies have uniformly identified a high baseline systolic ABP as a predictor for good BP response after RDN, but this phenomenon is unspecific and known as law of initial value (Wilder’s principle) [24, 26–34]. In our study we focused on the importance of skin Na content, assessed by Na-MRI, in 58 patients with TRH as a potential predictor of the BP response after RDN. The skin, like the muscles, is an important storage of extracellular Na content [16]. Na accumulation in tissue may exaggerate hypertrophy of the myocardial and vascular smooth muscle cells and thereby augment HTN associated complications [9, 16]. It is known that patients with TRH achieve a greater reduction in BP by restricting salt compared to other hypertensive patients and that patients with TRH are more sensitive to salt intake [35]. However, the effect of RDN on sodium homeostasis is not well understood. Ott et al. performed Na-MRI in 41 patients who had underwent RDN and observed no change in tissue Na content at the second examination after 6 months, in accordance with the results of the current study [18]. In contrast, a post-hoc analysis of 137 patients showed an increase in urinary Na in patients with TRH 6 months after RDN, but the reliability of the measurements were questionable as they were based on the Kawasaki formula and spot urine [36]. Experimental animal studies have also shown an increased Na excretion in the acute stage after RDN and a decrease in Na excretion after renal sympathetic nerve stimulation [37–39].

In this study we focused on a potential role of Na as a predictor of the BP drop after RDN. We restricted our analysis to ABP measurements following the current consensus statement [11, 12]. According to the change of systolic 24-h, day-time and night-time ABP 6 months after RDN we splitted the study cohort into responders and non-responders by the respective median drop in ABP. We found patients with low skin Na content to be more likely to respond to RDN than those with high baseline skin Na. This result persisted after adjustment for baseline 24-h systolic BP and were also found after separating the study cohort according to median decrease of the following BP parameters: 24-h ABP, day-time ABP and night-time ABP. With respect to muscle Na content, a lower content tended to be related to better ABP response. Thus, baseline skin Na content (and to lesser extent muscle sodium content) emerged as a predictor for systolic ABP response to RDN in addition to and independent from baseline systolic ABP. High tissue sodium was observed in several diseased populations, such as in patients with T2D, hypertension Conn’s syndrome and CKD [17, 23, 40, 41]. We previously showed that high tissue sodium content is linked to hypertrophic vascular remodeling and to left ventricular hypertrophy [40, 42]. Thus, we assume that patients with a higher skin Na content may be in a more advanced stage of arterial hypertension with greater extent of hypertrophic vascular remodeling and are therefore less likely to respond to renal denervation.

Our findings also suggest that gender, LDL cholesterol, aldosterone-antagonist medication, 24-h, day-time and night-time heart rate may play a role in determining BP response to RDN. Böhm et al. identified in the SPYRAL HTN-OFF MED pivotal trial a high baseline heart rate to determine the effectiveness of RDN [43]. In contrast, Esler et al. found no correlation between HR and renal sympathetic activation but only between HR and cardiac sympathetic activation [44]. It was previously shown that RDN reduces cardiac sympathetic activity [45]. We identified 24-h, day-time and night-time HR to be a predictive parameter for good BP response after RDN and this result remained significant after adjustment for baseline 24-h systolic ABP. However, we could not identify any correlation between 24-h, day-time or night-time heart rate and BP response. In addition, we identified patients with aldosterone-antagonist medication use to have a better BP response to RDN. Interestingly aldosterone-antagonist medication has also been identified as a predictor in the Symplicity HTN 3 trial [27]. In accordance, a single-center trial identified aldosterone-antagonist medication in patients with TRH to be predictive for better BP response after RDN [46]. However, other studies could not identify any relationship between aldosterone-antagonist medication and BP response after RDN [47, 48]. We also assessed correlation between aldosterone-antagonist medication use and BP response but did not observe a correlation.

Despite identifying that female patients were more likely in the responder group, we did not find any correlation between sex and BP response.

Several studies also analyzed vascular parameters to identify potential predictors for BP response after RDN. Weber et al. identified pulsatile hemodynamics such as augmentation index, augmentation pressure and estimated aortic pulse wave velocity to be potential predictors for BP response after RDN [49]. In accordance, other studies analyzed the influence of invasively measured pulse wave velocity on BP response after RDN and showed that patients with lower pulse wave velocity at baseline were more likely to be responders to RDN [50, 51]. Additionally, Fengler et al. identified cardiac magnetic resonance assessment of central and peripheral vascular function as a potential predictor of RDN [52]. In accordance, we previously identified lower baseline central pulse pressure (measured non-invasively with SphygmoCor™ device), indicative for the degree of arterial stiffening, to be a predictive value for better BP response after RDN [53].

Despite BP reduction, RDN may have pleiotropic effects. We defined non-responders and responders only by their BP change. However, there may be positive effects of RDN beyond BP reduction also in patients defined as non-responders.

Our study has several limitations. It is a single-center and post-hoc study with a small sample size with a limited follow-up of 6 months. However, 24-h, day-time and night-time systolic BP reduction demonstrated consistent results, with low skin Na being related to greater BP reduction after RDN. Though not significant, a similar signal has been found for baseline muscle content even after adjustment for baseline 24-h ABP. Nevertheless, only prospective studies with longer follow-up are able to corroborate our observations.

## Conclusion

Our findings suggest that patients with low skin Na have a greater BP reduction after RDN, independent of baseline ABP. Utilizing Na-MRI prior to RDN might provide a valuable tool in selecting patients with TRH that have a BP reduction after RDN above the median and thereby being particularly suitable for the procedure.

## Data Availability

The datasets used and analyzed for this register are available from the corresponding author on reasonable request.
